# A miRNA screening identifies miR-192-5p as associated with response to azacitidine and lenalidomide therapy in myelodysplastic syndromes

**DOI:** 10.1186/s13148-023-01441-9

**Published:** 2023-02-20

**Authors:** Sara Mongiorgi, Alessia De Stefano, Stefano Ratti, Valentina Indio, Annalisa Astolfi, Irene Casalin, Andrea Pellagatti, Stefania Paolini, Sarah Parisi, Michele Cavo, Andrea Pession, James A. McCubrey, Pann-Ghill Suh, Lucia Manzoli, Jacqueline Boultwood, Carlo Finelli, Lucio Cocco, Matilde Y. Follo

**Affiliations:** 1 Cellular Signalling Laboratory, Department of Biomedical and Neuromotor Sciences, University of Bologna, Via Irnerio 48, 40126 Bologna, Italy; 2 “Giorgio Prodi” Cancer Research Center, University of Bologna, Via Massarenti 9, 40138 Bologna, Italy; 3 Department of Medical and Surgical Sciences, University of Bologna, Via Massarenti 9, 40138 Bologna, Italy; 4 Blood Cancer UK Molecular Haematology Unit, Nuffield Division of Clinical Laboratory Sciences, Radcliffe Department of Medicine, University of Oxford, and Oxford BRC Haematology Theme, Oxford, OX3 9DU UK; 5 IRCCS - Azienda Ospedaliero-Universitaria di Bologna, Institute of Hematology “ L. e A. Seràgnoli”, University of Bologna, Via Massarenti 9, 40138 Bologna, Italy; 6 IRCCS Azienda Ospedaliero-Universitaria di Bologna, Division of Pediatrics, University of Bologna, Via Massarenti 9, 40138 Bologna, Italy; 7 Department of Microbiology and Immunology, Brody School of Medicine, East Carolina University, Greenville, NC 27858 USA; 8 Korea Brain Research Institute, Daegu, 41062 South Korea

**Keywords:** miRNA profiling, Myelodysplastic syndromes, BCL2, Azacitidine, Lenalidomide

## Abstract

**Background:**

miRNAs are small non-coding RNAs that regulate gene expression and are linked to cancer development and progression. miRNA profiles are currently studied as new prognostic factors or therapeutic perspectives. Among hematological cancers, myelodysplastic syndromes at higher risk of evolution into acute myeloid leukemia are treated with hypomethylating agents, like azacitidine, alone or in combination with other drugs, such as lenalidomide. Recent data showed that, during azacitidine and lenalidomide therapy, the concurrent acquisition of specific point mutations affecting inositide signalling pathways is associated with lack or loss of response to therapy. As these molecules are implicated in epigenetic processes, possibly involving miRNA regulation, and in leukemic progression, through the regulation of proliferation, differentiation and apoptosis, here we performed a new miRNA expression analysis of 26 high-risk patients with myelodysplastic syndromes treated with azacitidine and lenalidomide at baseline and during therapy. miRNA array data were processed, and bioinformatic results were correlated with clinical outcome to investigate the translational relevance of selected miRNAs, while the relationship between selected miRNAs and specific molecules was experimentally tested and proven.

**Results:**

Patients’ overall response rate was 76.9% (20/26 cases): complete remission (5/26, 19.2%), partial remission (1/26, 3.8%), marrow complete remission (2/26, 7.7%), hematologic improvement (6/26, 23.1%), hematologic improvement with marrow complete remission (6/26, 23.1%), whereas 6/26 patients (23.1%) had a stable disease. miRNA paired analysis showed a statistically significant up-regulation of miR-192-5p after 4 cycles of therapy (vs baseline), that was confirmed by real-time PCR analyses, along with an involvement of BCL2, that was proven to be a miR-192-5p target in hematopoietic cells by luciferase assays. Furthermore, Kaplan–Meier analyses showed a significant correlation between high levels of miR-192-5p after 4 cycles of therapy and overall survival or leukemia-free survival, that was stronger in responders, as compared with patients early losing response and non-responders.

**Conclusions:**

This study shows that high levels of miR-192-5p are associated with higher overall survival and leukemia-free survival in myelodysplastic syndromes responding to azacitidine and lenalidomide. Moreover, miR-192-5p specifically targets and inhibits BCL2, possibly regulating proliferation and apoptosis and leading to the identification of new therapeutic targets.

**Supplementary Information:**

The online version contains supplementary material available at 10.1186/s13148-023-01441-9.

## Background

Epigenetic processes, such as those regulated by miRNAs, modulate gene expression and affect cell proliferation and differentiation [1]. miRNAs are non-coding small RNAs that form key molecular components of the cell and have the important ability to modulate gene expression. Consequently, any miRNA dysregulation can lead to clinical manifestation of disease [2]. Cancer development depends on miRNA expression, so that miRNA profiles are now investigated as new prognostic factors or therapeutic new perspectives in several human cancers, like myelodysplastic syndromes (MDS) and acute myeloid leukemia (AML) [3, 4].

To date, the only currently approved treatments for higher-risk MDS are allogeneic stem cell transplantation and hypomethylating agents, alone or in combination with novel drugs [5]. Azacitidine (AZA) is indeed a standard first-line therapy in high-risk MDS. Its combination with venetoclax, a BCL2 inhibitor, is now under evaluation [6], while the association between AZA and lenalidomide (LEN) has been already tested in clinical trials [7, 8]. At a molecular level, further data on AZA + LEN therapy are needed, to better understand their pathogenetic role and the mechanisms of resistance to therapy. Of note, recent data showed that the acquisition of three specific point mutations on PI3KCD, PLCG2 and AKT3 genes can be associated with loss or lack of response to AZA + LEN [9].

Inositide signalling is implicated in cell metabolism [10–12], and phosphoinositide-specific phospholipase C (PI-PLC) beta1 and gamma1, besides playing a role in cell proliferation, differentiation and disease [13–17], are specifically involved in the progression of MDS to AML [18–22] and are a target for miRNA regulation[23–25].

miR-192–5p, belonging to the miR-192/215 family, is a conserved tumor and leukemia-related miRNA [26]. Abnormal expression of miR-192 has been observed in several human cancers, and down-regulation of serum miR-192 in pediatric AML patients not only correlated with aggressive clinical features, but it could also serve as an independent prognostic indicator for both overall survival and event-free survival [27]. At a molecular level, miR-192 has a tumor-suppressive role in cancer, via regulation of BCL2, TP53 and TGF-β signalling [28–30], as well as inhibition of CCNT2 in leukemia [31]. Therefore, miR-192 expression can significantly suppress cell proliferation and induce G0/G1 cell cycle arrest of AML cells [32]. Even the down-regulation of miR-21-5p can inhibit cell proliferation, induce apoptosis and cause G1 cell cycle arrest, through the reduction of AKT, CCND1 and BCL2 [33], while miR-224-5p is another miRNA that can target BCL2, as well as other genes (including RAC1, RAB10, AKT3 and p-ERK/ERK signalling), to regulate the growth of AML cells [34].

Here we further analyzed the role of AZA + LEN therapy in MDS, focusing on miRNA expression, with the aim to select some specific miRNAs and study their interactions with specific targets, such as BCL2. The modulation of miR-192-5p during therapy might indeed be associated with clinical outcome or therapy response, thus leading to the identification of new pathogenetic mechanisms and molecular targets.

## Results

### Patient outcomes

Between March 2013 and December 2017, 44 patients diagnosed with high-risk MDS were treated with a combination of azacitidine and lenalidomide (Eudract clinical protocol n. 2011-005322-22). 31 cases were clinically evaluable for response, and 3 patients showed a disease progression or hematologic improvement before the 4th cycle of therapy (T4) and were evaluated for response too, so that 34 cases were clinically evaluable for response. However, as the quality and quantity of cells available for each patient at both baseline (T0), T4 and the 8th cycle of therapy (T8) was critical, molecular analyses for this study were performed only on 26/34 samples (Table 1). The median follow-up of these 26 patients was 32 months (range 3–42 months), while their overall response rate, assessed by the revised IWG criteria [35], was 76.9% (20/26 cases): complete remission (CR, 5/26, 19.2%), partial remission (PR, 1/26, 3.8%), marrow CR (mCR, 2/26, 7.7%), hematologic improvement (HI, 6/26, 23.1%), HI + mCR (6/26, 23.1%), whereas 6/26 patients (23.1%) had a stable disease (Table 1). For our molecular analyses, we divided patients into three subgroups: responders (showing response within T4, and maintaining it at T8; *n* = 10), early losing response (showing response within T4 and losing it at T8; *n* = 10) and non-responders (never showing a response; *n* = 6).

**Table 1:** Clinical, hematologic, and cytogenetic characteristics of the MDS patients

	Age	Sex	Diagnosis		Screening	Karyotype [No. Metaphases with aberrration]	Clinical outcome	Total cycles	Duration of therapy (Months)	Time to first response (Cycles)	Duration of response (Months)	Survival (Months)	Time to AML Evolution (Months)	Cause of death	Clinical response’s subgroup for molecular analysis
			WHO	WPSS											
1^†^	67	M	RAEB-2	VERY-HIGH	25/03/2013	COMPLEX	SD	10	10			14	8	AML	NR
2^†^	67	F	RAEB-2	HIGH	02/04/2013	46, XX	CR	30	28	6	24	35	28	OVARIAN CANCER	R
3^†^	71	M	RAEB-2	HIGH	29/04/2013	47, XY, + 8	mCR	38	36	4	10	41	36	AML, INFECTION	R
4^†^	76	F	RAEB-2	HIGH	13/05/2013	46, XX	HI	8	8	4	5	12	9	AML, CACHEXIA	LR
5^†^	68	M	RAEB-1	HIGH	13/05/2013	COMPLEX	SD	9	9			14		PNEUMONIA, CARDIAC FAILURE	NR
6^†^	67	M	RAEB-2	HIGH	23/05/2013	46, XY	PR	10	10	2	6	30	10	AML	LR
7^†^	72	M	RAEB-1	HIGH	26/06/2013	46, XY, del (7) (q22;34) [20]; del (7q31) [18]	SD	8	8			22	20	CEREBRAL HEMORRHAGE	NR
8	82	M	RAEB-2	HIGH	28/06/2013	46, XY	CR	41	42	2	40	42			R
9	67	F	RAEB-1	HIGH	01/07/2013	47, XX, + 8	HI + mCR	12	18	2 (mCR); 5 (HI)	8 (HI); 16 (mCR)	42			R
10	73	F	RAEB-1	INT	10/07/2013	45, X, del X, del (20q)	HI + mCR	38	42	3 (HI); 4 (mCR)	35 (HI); 34 (mCR)	42			R
11^†^	75	F	RAEB-1	HIGH	10/07/2013	47, XX, + 8	HI	20	21	1	19	28	21	DISEASE PROGRESSION	R
12	76	M	RAEB-1	INT	22/07/2013	46, XY	HI + mCR	8	8	1	5 (HI); 6 (mCR)	38			LR
13^†^	70	M	RAEB-2	HIGH	29/08/2013	46, XY	CR	27	40	2	38	40			R
14^†^	72	F	RCMD-RS	HIGH	09/09/2013	COMPLEX	HI + mCR	13	14	2	11	16	15	AML, SEPSIS	LR
15^†^	68	M	RCMD	HIGH	03/10/2013	45, XY, del 7	HI	3	3	1	2	3		SUDDEN DEATH	NR
16^†^	66	F	RAEB-2	HIGH	15/10/2013	46, XX	SD	8	8			14	14	AML	NR
17^†^	48	F	RAEB-2	VERY-HIGH	6/11/2013	47, XY, + 8	CR	16	15	2	11	26		DISEASE PROGRESSION	R
18^†^	64	F	RAEB-1	INT	6/11/2013	46, XX	HI	10	10	5	3	12		PULMONARY CARCINOMA	LR
19^†^	66	M	RAEB-2	VERY-HIGH	15/11/2013	47, XY, + 8	HI	9	9	3	5	10		WORSENING OF CLINICAL CONDITIONS	LR
20^†^	75	F	RAEB-2	HIGH	22/11/2013	46, XX	HI	10	10	2	7	11	11	AML	LR
21^†^	83	M	RAEB-2	HIGH	04/02/2014	47, XX, + 8 [5]	SD	6	6			11	9	AML	NR
22^†^	71	F	RAEB-2	VERY-HIGH	17/02/2014	COMPLEX	CR	7	7	2	5	12	9	AML, SEPSIS	LR
23	72	M	RAEB-1	VERY-HIGH	14/4/2014	COMPLEX	HI + mCR	11	11	4 (HI + mCR)	6 (HI)	32			R
24^†^	69	M	RAEB-2	HIGH	19/5/2014	46, XY	mCR	6	6	2	3	16	13	AML	NR
25	70	M	RAEB-1	HIGH	19/5/2014	46, XY, del (7q31)	SD	7	9			31			NR
26^†^	78	M	RAEB-2	HIGH	19/8/2014	46, XY	HI + mCR	17	16	2 (HI); 2 (mCR)	14 (HI); 14 (mCR)	17	17	UNKNOWN	R

*WHO* World Health Organization, *WPSS* WHO prognostic scoring system, *RAEB* Refractory anemia with excess of blasts, *RCMD* Refractory cytopenia with multilineage dysplasia, *RCMD-RS* RCMD with ring sideroblasts ≥ 15%, *SD* Stable disease, *CR* Complete remission, *mCR* Marrow complete remission, *PR* Partial remission, *HI* Hematologic improvement, *R* Responder patients (showing response within the 4th cycle of therapy, T4, and maintaining it at the 8th cycle, T8); *LR* Patients early losing response (showing response within T4 and losing it at T8); *NR* Non-responder patients (never showing a response)

† Patient deceased

### miRNAs deregulation in MDS during azacitidine and lenalidomide therapy

**Fig. 1:**
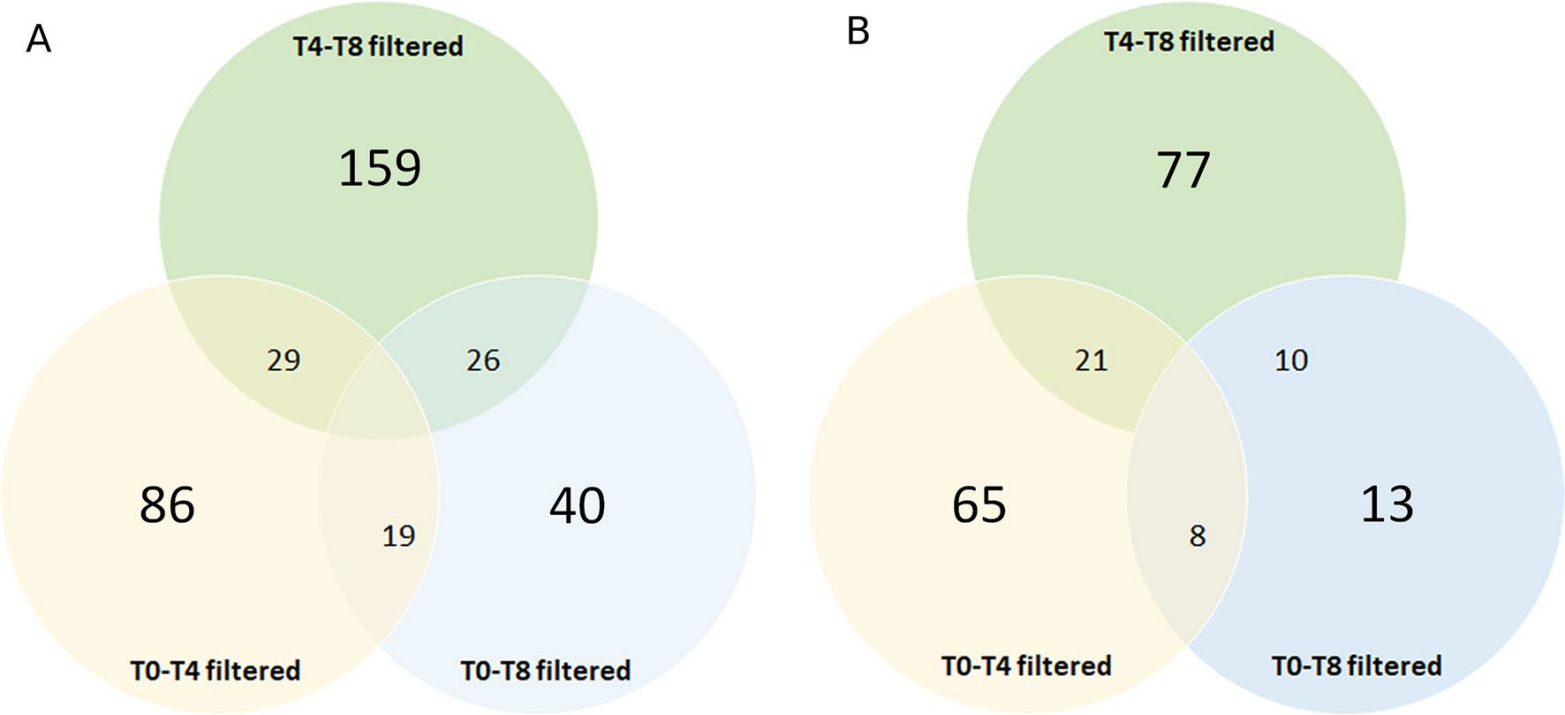
Clustering analysis of dysregulated miRNAs in MDS patients during AZA + LEN therapy. **A** Venn diagram displaying the overlap among all differentially expressed miRNAs (including miRNA precursors and short RNAs not belonging to the miRNA family) at baseline (T0), the 4th (T4) and the 8th (T8) cycle of AZA + LEN therapy. Data are filtered by setting the statistical significance at *p* value < 0.05 and abs(logFC) > 0.5. **B** Venn diagram displaying the overlap among differentially expressed mature miRNAs at baseline (T0), the 4th (T4) and the 8th (T8) cycle of AZA + LEN therapy. Data are filtered by setting the statistical significance at *p* value < 0.05 and abs(logFC) > 0.5

By the Affymetrix GeneChip miRNA 4.0 array, 6658 human probes were analyzed in bone marrow (BM) mononuclear cells (MNCs) obtained from MDS patients treated with AZA + LEN therapy, including miRNA precursors and short RNAs not belonging to the miRNA family. Data are reported in Additional file 1: Tables S1-S6. By setting the statistical significance at *p* value < 0.05 and abs(logFC) > 0.5, 134/6658 sequences resulted to be differentially expressed between T0 and T4 (i.e., 94 mature miRNAs, 4 snoRNAs, 4 HAcaBox, 4 stem-loop and 28 CDBox); 214/6658 between T4 and T8 (i.e., 108 miRNAs, 20 snoRNAs, 10 HAcaBox, 19 stem-loop, 47 CDBox and 10 5.8s rRNA) and 85/6658 between T0 and T8 (i.e., 31 miRNAs, 6 snoRNAs, 6 HAcaBox, 7 stem-loop, 32 CDbox, 2 scaRna and 1 Spike-in control) (Fig. 1A). Then, we focused on the mature miRNAs overlapping among the three groups, excluding them, and including in the subsequent analyses only mature miRNAs belonging specifically to one subgroup. This shortened the list of differentially expressed miRNAs to 65 miRNAs (61 up- and 4 down-regulated) between T0 and T4, 77 miRNAs (57 up- and 20 down-regulated) between T4 and T8; and 13 miRNAs (11 up- and 2 down-regulated) between T0 and T8 (Fig. 1B). Among the newest identified and altered miRNAs involved in MDS pathogenesis or AML evolution [36–38], miR-15a-5p, miR-22, miR-378-3p were significantly increased only at T4, as compared to T0 (miR-15-a-5p: *p* = 0.01208; miR-22: *p* = 0.0345; miR-378-3p: *p* = 0.03115).

As for miRNAs involved in inositide signalling pathways, we analyzed the Affymetrix output data through miRPathDB 2.0 and KEGG software. Considering only miRNAs showing a Log2(FC) > 0.5 or Log2(FC) <—0.5 and included in both databases, four sequences were identified as statistically significant: miR-192-5p and miR-21-5p (up-regulated at T4, *p* = 0.00192 and *p* = 0.00248 respectively), miR-224-5p (down-regulated at T8 as compared with T4, *p* = 0.00787) and hsa-let-7f-2-3p (down-regulated at T8 as compared with T0, *p* = 0.00322). As only the first three miRNAs can be linked to PI3K/Akt or PLC signalling [39], we subsequently focused only on these three miRNAs.

### Validation of microarray data: gene expression of miR-192-5p, miR-21-5p and miR-224-5p

**Fig. 2:**
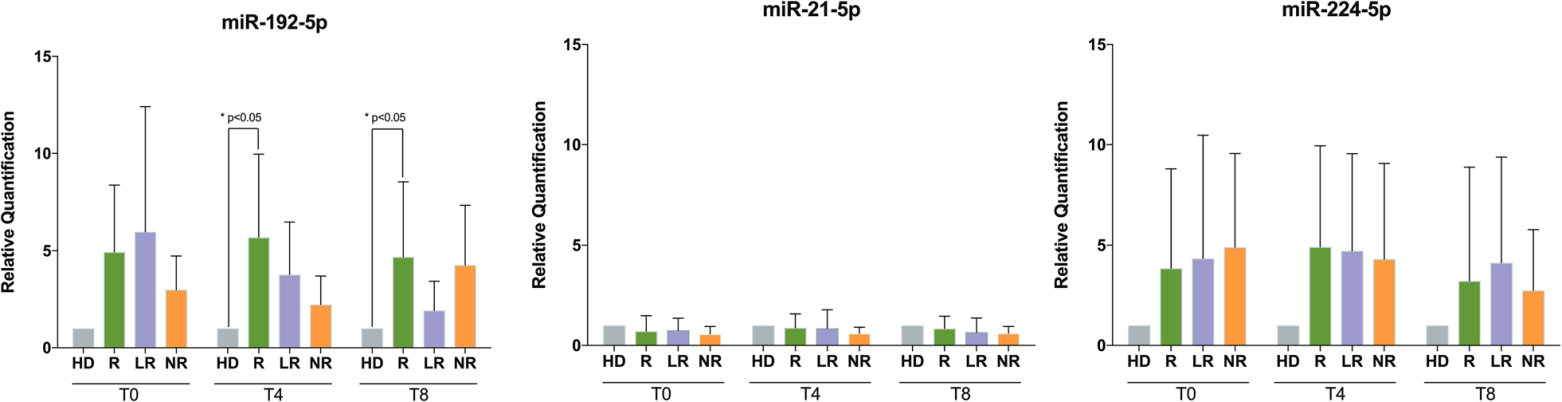
miRNA expression in MDS patients. **A** miR-192-5p expression in BMMNCs obtained from MDS patients at baseline (T0), after 4 (T4) and 8 (T8) cycles of AZA + LEN therapy. Responder patients (R) show high levels of miR-192-5p, as compared to healthy subjects (HD), at T0, T4 and T8. During treatment, they showed a slight increase at T4 (vs T0), followed by a decrease at T8 (vs T4). Even patients losing response (LR) show high miR-192-5p levels at T0 and a reduction during therapy, while non-responder patients (NR) show the lowest level of miR-192-5p at T0 (although being higher than HD), a slight reduction at T4 and an increase at T8. **B** miR-21–5-p expression in MDS patients at baseline (T0), after 4 (T4) and 8 (T8) cycles of AZA + LEN therapy. All patients’ subgroups [responders (R), losing response (LR), and non-responders (NR)] show similar and constant levels of miR-21-5p, that are not statistically different from healthy subjects (HD) and are similar throughout the treatment. **C** miR-224-5p expression in MDS patients at baseline (T0), after 4 (T4) and 8 (T8) cycles of AZA + LEN therapy. All patients’ subgroups [responders (R), losing response (LR), and non-responders (NR)] show higher levels of miR-224-5p at T0, T4 and T8, as compared to healthy subjects (HD), although not reaching statistical significance. R and LR patients show a slight increase at T4 (vs T0) and a slight reduction at T8 (vs T4), while NR show a small decrease at both T4 and T8, as compared with T0. All data are represented as mean ± SD. Each experiment was repeated at least three times, with at least six replicates for each treatment condition and patients’ subgroup. *p < 0.05 versus HD, by one-way ANOVA using Bonferroni multiple comparisons in GraphPad Prism 5

As Fig. 2 shows, the amount of miR-192-5p was always increased in MDS cells from all groups at T0, as compared with healthy subjects, and during therapy. This showed significant similarity with the gene expression dataset GSE107400, obtained from the Gene Expression Omnibus (GEO) database. Similar to our findings, the expression levels of miR-192 in that population were indeed higher in MDS patients than in healthy subjects [40] (Additional file 2: Figure S1).

In our cohort, only at T4 and T8 there was a statistically significant difference of miR-192-5p amount in responder patients compared with healthy subjects (T4: Student’s *t* test, *p* < 0.05, 95% CI − 7.72 to 0.69; T8: Student’s *t* test, *p* < 0.05, 95% CI − 6.93 to − 0.42), while the changes between the other subgroups and healthy subjects did not reach statistical significance. Comparing the level of miR-192-5p within the subgroups of patients during the time course, the expression in responders at baseline and during therapy was always higher than in MDS patients early losing response and in non-responders, with a slight increase at T4 in responders, as compared with T0 and T8 within the same patients’ subgroup. On the contrary, the expression of miR-21-5p was almost constant and not statistically different from healthy subjects nor during time course in all groups analyzed. Finally, even though miR-224-5p expression was increased in MDS cells at T0 and during the therapy in all samples, the differences among the samples and the time course were not statistically significant.

### Gene expression of BCL2, PLCB1 and PLCG1

**Fig. 3:**
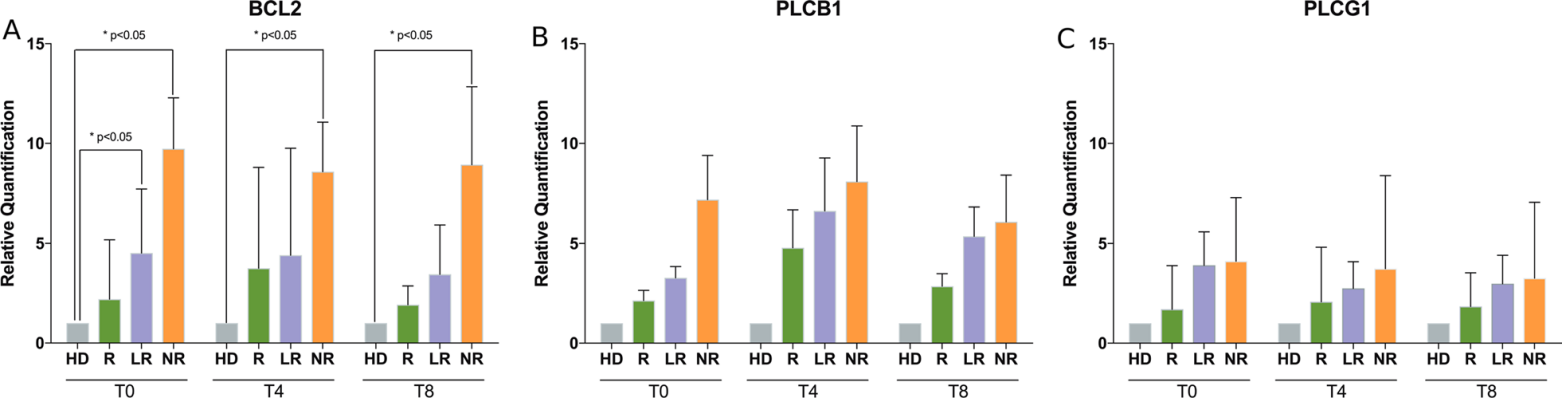
Gene expression of BCL2 and PLC genes in MDS patients. **A** BCL2 gene expression in cells obtained from MDS patients at baseline (T0), after 4 (T4) and 8 (T8) cycles of AZA + LEN therapy. Responder patients (R) show high levels of BCL2 at T0, T4 and T8, as compared to healthy subjects (HD), increasing levels at T4 (vs T0) and a reduction at T8 (vs T4). Patients losing response (LR) show high levels at T0 (statistically significant), T4 and T8 as compared to healthy subjects (HD), a constant amount at T4 (vs T0) and a slight reduction at T8 (vs T4). Non-responder (NR) patients show high levels of BCL2 at T0, T4 and T8, whose difference with healthy subjects (HD) is always statistically significant. During treatment, the amount of BCL2 in NR is slightly reduced. **B** PLCB1 expression in MDS patients at baseline (T0), after 4 (T4) and 8 (T8) cycles of AZA + LEN therapy. All patients’ subgroups [responders (R), losing response (LR), and non-responders (NR)] show higher levels of PLCB1, that are not statistically different from healthy subjects (HD). At T4, all patients’ subgroups show a slight induction (as compared with T0) and a subsequent reduction at T8 (vs T4), with LR and NR subgroups always showing higher levels than R. **C** PLCG1 expression in MDS patients at baseline (T0), after 4 (T4) and 8 (T8) cycles of AZA + LEN therapy. All patients’ subgroups [responders (R), losing response (LR), and non-responders (NR)] show higher levels of PLCG1, that are not statistically different from healthy subjects (HD). At T4, LR and NR subgroups show a reduction, while R display a slight increase, as compared with T0 (not statistically significant). At T8, R and NR show a reduction and LR display a slight increase, as compared with T4 (not statistically significant). All data are represented as mean ± SD. Each experiment was repeated at least three times, with at least six replicates for each treatment condition and patients’ subgroup. *p < 0.05 versus HD, by one-way ANOVA using Bonferroni multiple comparisons in GraphPad Prism 5

As also observed in the GEO datasets GSE4619 and GSE19429 [41, 42], even in our case series the amount of BCL2 gene expression was increased at baseline in MDS, as compared with healthy subjects (Additional file 2: Figure S1). Moreover, our MDS patients at higher risk showed increased baseline levels of BCL2, as also seen in other MDS studies, though these studies were focused on BCL2 protein [43–45]. In our cohort, the amount of BCL2 gene expression was significantly increased in MDS cells extracted from patients early losing response at T0 (Student’s *t* test, *p* < 0.05, 95% CI − 6.98 to − 0.05) and in non-responders at both T0, T4 and T8, as compared with healthy subjects (T0: Student’s *t* test, *p* < 0.05, 95% CI − 13.69 to − 3.76; T4: Student’s *t* test, *p* < 0.05, 95% CI − 14.2 to − 0.96; T8: Student’s *t* test, *p* < 0.05, 95% CI − 14.05 to − 1.79), whereas the increased amount in responders at T0, T4 and T8, compared with healthy subjects, never reached statistical significance (Fig. 3A). Even during the time course (T0 vs T4, T4 vs T8, T8 vs T0), BCL2 expression changes in all patients’ subgroups (responders, early losing response and non-responders) did not reach statistically significant differences.

As for PI-PLC genes, PLCB1 and PLCG1 showed an altered expression during therapy, although not statistically significant. In responders, starting from the lowest baseline levels, PLCB1 displayed an increase at T4 and a reduction at T8 (Fig. 3B), while PLCG1 levels were maintained during the time course (Fig. 3C). In contrast, in patients losing response and in non-responders, the expression of both PLCB1 and PLCG1 was almost constant or slightly decreased between T4 and T8, although never reaching the levels of MDS responders nor the healthy subjects (Fig. 3B, C).

### miRNA target validation: luciferase assay

It is not yet clear how many sites of interaction for miR-192-5p occur at the 3′-UTR of BCL2, as our bioinformatic analyses on biological target prediction (by using STarMirDB [46], miRBase [47], TargetScan [48] and miRDB [49] software and databases), found out 3′-UTR seed sequences only for BCL-associated proteins, such as BIM, but not directly with BCL2. Evidences of interactions with BCL2 came from experimentally verified or miRNA predicted target resources [50–52]. Therefore, to further investigate whether miR-192-5p really targets 3′-UTR BCL2, THP-1 cells were co-transfected with a 3′-UTR BCL2 reporter vector and miR-192-5p mimics, as well as a positive control and a scrambled (negative) control (Fig. 4). After 24 h from co-transfection of 3′-UTR BCL2 with miR-192-5p mimics, the relative luciferase ratio (FLuc/RLuc) was 26% (*p* = 0.02), as compared to cells co-transfected with negative control, thus showing similarities with the positive control (25%, *p* = 0.01).

**Fig. ﻿4:**
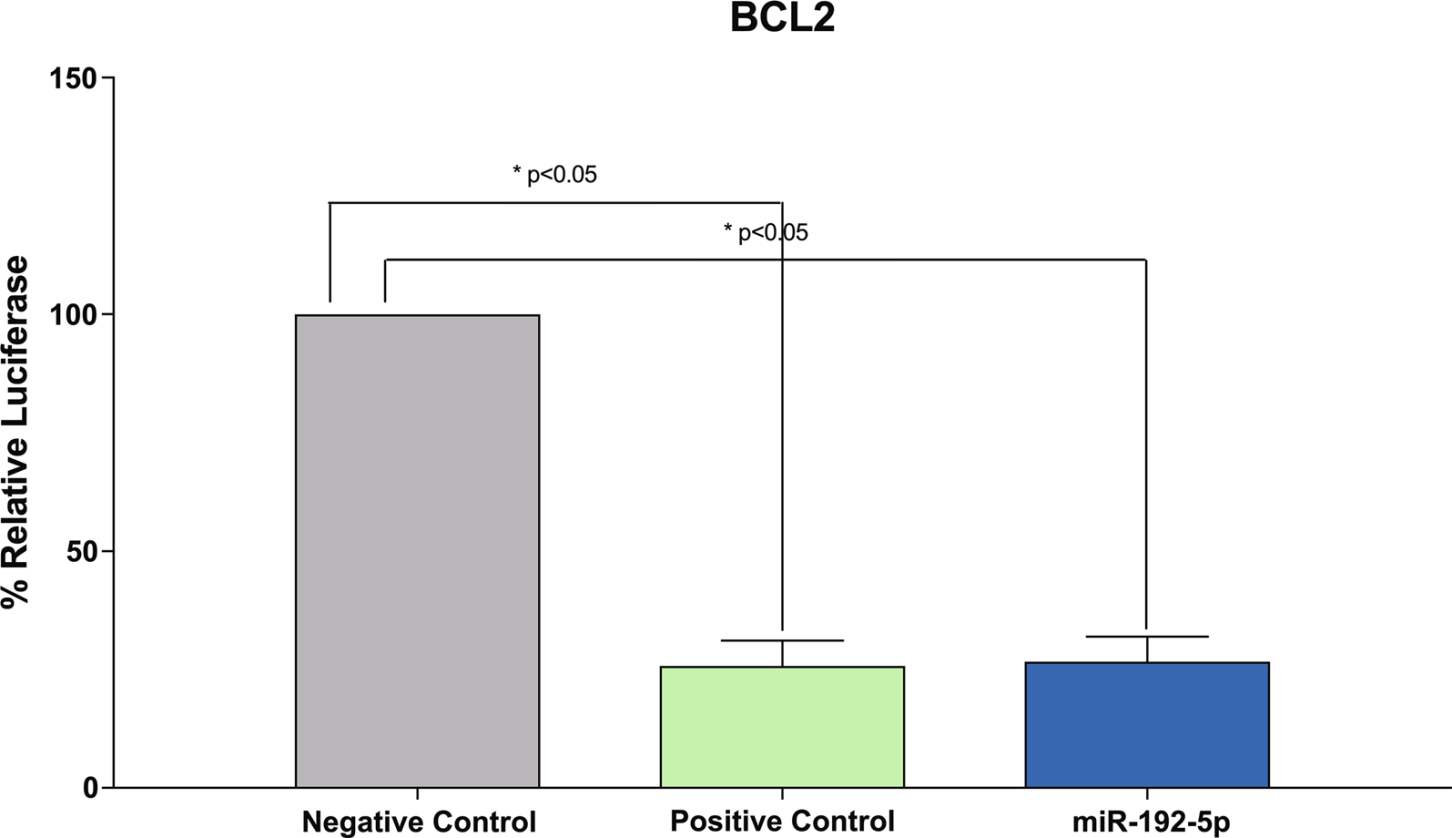
miR-192-5p directly targets BCL2 Promoter Sequence. THP-1 cells were transduced with miR-192-5p mimics, positive control, or empty vector (negative control) and a 3′-UTR BCL2 reporter vector. Results are expressed as the relative ratio of Firefly luciferase activity to Renilla luciferase activity using the negative control as reference. Data represent at least three biological replicates with three technical replicates and are shown as mean ± SD. *p < 0.05 versus negative control, by one-way ANOVA using Dunnett’s multiple comparisons in GraphPad Prism 5

### miRNA prognostic relevance: survival analyses

**Fig. 5:**
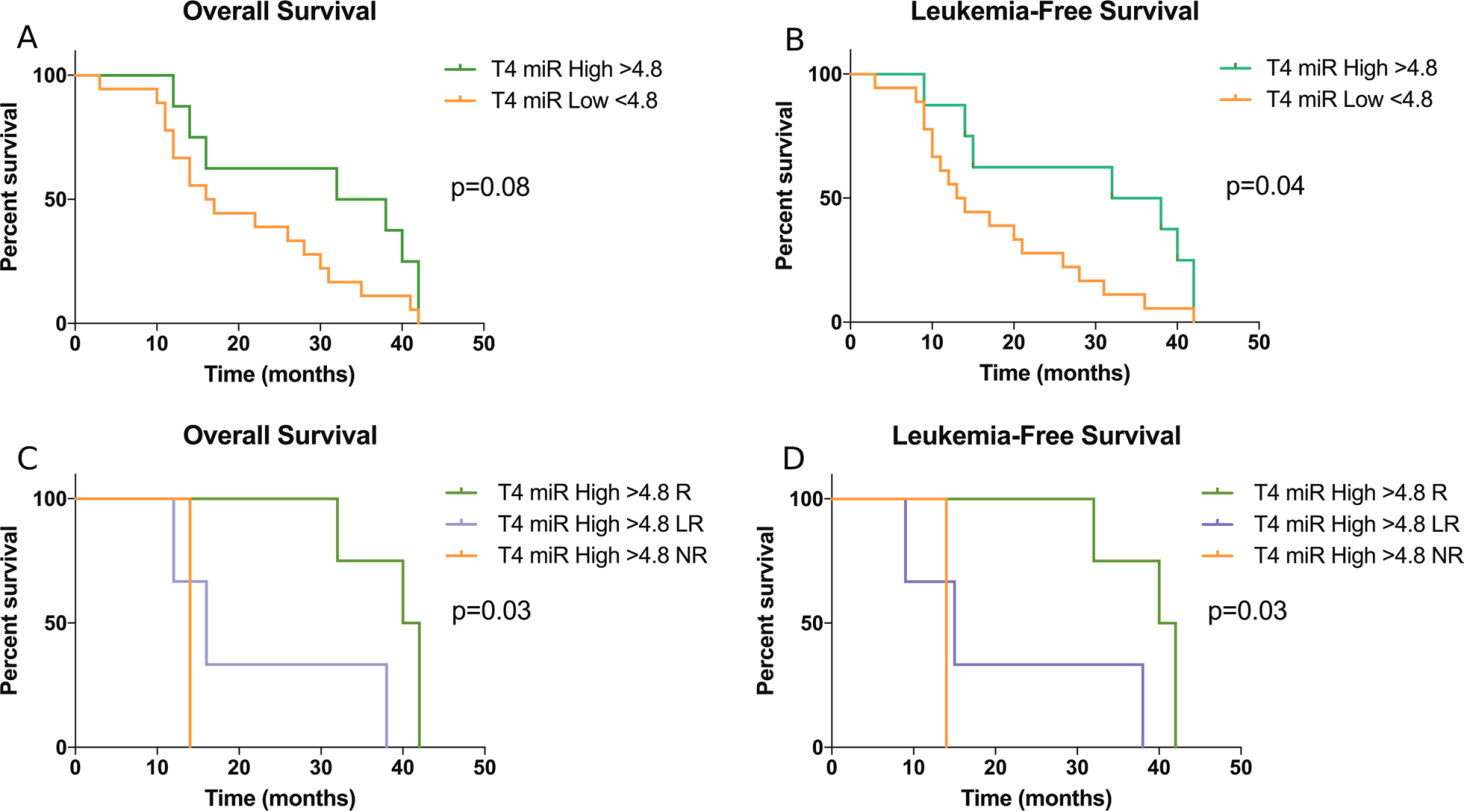
Prognostic significance of miR-192-5p in MDS patients at the 4th cycle of AZA + LEN treatment. **A** Kaplan–Meier curve comparing overall survival in twenty-six patients showing high (> 4.8) and low (< 4.8) miR-192-5p level at the 4th cycle of therapy (T4). P values were determined using a log-rank test. **B** Kaplan–Meier curve comparing leukemia-free survival in twenty-six patients showing high (> 4.8) and low (< 4.8) miR-192-5p level at the 4th cycle of therapy (T4). *p* values were determined using a log-rank test. **C** Kaplan–Meier curve comparing overall survival in MDS patients showing high (> 4.8) miR-192-5p level at the 4th cycle of therapy (T4) and divided according to the response to therapy in responders (R), losing response (LR) and non-responders (NR). *p* values were determined using a log-rank test. **D** Kaplan–Meier curve comparing leukemia-free survival in MDS patients showing high (> 4.8) miR-192-5p level at the 4th cycle of therapy (T4) and divided according to the response to therapy in responders (R), losing response (LR) and non-responders (NR). *p* values were determined using a log-rank test

ROC analysis was firstly performed to correlate the expression of miR-192-5p with AZA + LEN response. The area under the curve (AUC) results showed that the expression of miR-192-5p in MDS was globally higher and statistically different from the healthy subjects (AUC = 0.77, 95% CI + 0.62 to + 0.92, *p* = 0.013). With a cut-off value for miR-192-5p expression in MDS set at 4.8, the test sensitivity was 0.69 (95% CI + 0.48 to + 0.85) and the specificity was 0.90 (95% CI + 0.55 to + 0.99). Therefore, considering this level of miR-192-5p expression in MDS as cut-off, we studied the association between high (> 4.8) or low (< 4.8) levels of miR-192-5p and OS/LFS in our MDS patients. As reported in Fig. 5A, the association between high miR-192-5p levels at T4 and OS was close to significant: 35 versus 16.5 months with 95% CI + 1.68 to + 2.55, *p* = 0.08; HR = 0.47 with 95% CI + 0.20 to + 1.21, while the association between high miR-192-5p levels at T4 and LFS was statistically significant (Fig. 5B): 35 versus 13.5 months with 95% CI + 2.15 to + 3.02, *p* = 0.04; HR = 0.40 with 95% CI + 0.16 to + 0.96. Moreover, as reported in Fig. 5C, D, there was a significant trend between high miR-192-5p levels at T4 and both OS and LFS, and the correlation was stronger (*p* = 0.03) in case of responders, as compared with patients losing response and non-responders. On the contrary, the association between high miR-192-5p levels at T0 or T8 and OS or LFS did not reach statistical significance (miR-192-5p at T0 and OS: *p* = 0.17; miR-192-5p at T8 and OS: *p* = 0.39; miR-192-5p at T0 and LFS: *p* = 0.14; miR-192-5p at T8 and LFS: *p* = 0.51).

## Discussion

Hypomethylating therapy in MDS suffers from the fact that it requires some cycles to evaluate the response, must be administered until response is detectable and sometimes the response is short-lived. The combination of AZA + LEN has proven effectiveness (although it has not been shown to be significantly more effective than AZA alone in terms of OS), but the molecular mechanisms underlying this approach are still not well known. Dysregulation of miRNAs has been shown to be important in the pathogenesis of MDS and can also have prognostic and therapeutic relevance.

Here, we studied the effect of AZA + LEN therapy on miRNA expression, finding out that the treatment can indeed induce specific miRNA alterations. In particular, the analysis of miRNA profiling at baseline, after the 4th and after the 8th cycle of AZA + LEN therapy showed several miRNAs differentially expressed (up-or down-regulated). After a bioinformatic analysis, that allowed to detect specific clusters of expression, we found out three miRNAs particularly attractive: miR-192-5p and miR-21-5p, that were up-regulated after 4 cycles of therapy, and miR-224-5p, which was specifically down-regulated between the 4th and the 8th cycle. Interestingly, all these three miRNAs are associated with PI3K/AKT or PLC signalling and can, theoretically, target BCL2.

We then validated the microarray analyses, determining a good correlation between miRNA profiling and gene expression only for miR-192-5p, whose expression was always increased in MDS cells at baseline and during therapy, as compared with healthy subjects. More interestingly, at the 4th and 8th cycle of AZA + LEN therapy there was a statistically significant induction of miR-192-5p expression only in responder patients, while the increase did not reach statistical significance in both patients early losing response and non-responders, as compared with healthy subjects. As for BCL2 expression, it was particularly increased in MDS cells extracted from patients losing response and in non-responders at baseline and during AZA + LEN therapy, as compared with healthy subjects. On the contrary, in responder patients, BCL2 gene expression, although being higher than the healthy subjects, was always lower than in the other subgroups and even showing reducing levels at the 8th cycle of AZA + LEN therapy. As for PI-PLC genes, none of them were strictly correlated with the expression of any miRNA analyzed nor with response to AZA + LEN therapy, although PLCB1 modulation was slightly increased in responders.

Considering both miR-192-5p and BCL2 gene expression, we therefore speculated a correlation between these two molecules even in our experimental model, that was confirmed by dual luciferase assay results. Indeed, we showed that miR-192-5p specifically targets BCL2 promoter, and the low BCL2 gene expression in our MDS responder patients hints at a suppressive role of miR-192-5p, possibly correlating with inhibition of proliferation in this patients’ subgroup. Stemming from these data, we further analyzed the prognostic relevance of miR-192-5p in our MDS cohort, finding out that high miR-192-5p levels at the 4th cycle of AZA + LEN therapy are significantly associated with higher OS and LFS in responder patients and can be used to better stratify patients in responders, losing response and non-responders.

## Conclusions

Overall, our data show that miR-192-5p targets BCL2 and its expression might correlate with response to AZA + LEN therapy, possibly leading to several new lines of investigation relevant for both an improvement of patients’ stratification during AZA + LEN therapy and a better comprehension of miRNA regulation in MDS.

## Materials and methods

### Patient characteristics

Bone marrow (BM) and peripheral blood (PB) samples were obtained from 26 higher risk MDS patients who had given informed consent according to the Declaration of Helsinki (Table 1). All samples came from several Italian hematological centers and were centralized at the IRCCS—Azienda Ospedaliero-Universitaria di Bologna, Institute of Hematology “L. e A. Seràgnoli”, University of Bologna, Bologna, Italy. Further details can be found in the Additional File 3: Supplementary materials and methods.

### Patient treatment and evaluation of response

Patients were treated with AZA (75 mg/m2/die for 7 days every 28 days) and LEN (10 mg/day, days 1–21 or 8–21, orally) every 4 weeks, according to the Eudract clinical protocol n. 2011-005322-22. The response to treatment and the clinical outcome were evaluated according to the revised International Working Group (IWG) response criteria [35]. Further details can be found in the Additional File 3: Supplementary materials and methods .

### Isolation of mononuclear cells, total RNA extraction and gene expression

For in vitro experiments, BM and PB mononuclear cells (MNCs) were isolated by Ficoll-Paque density-gradient centrifugation (Amersham Biosciences, Uppsala, Sweden), according to the manufacturer’s instructions and as described elsewhere [53]. Analyses on MDS were carried out at the time of diagnosis and during the therapy and compared with MNCs isolated from healthy subjects. Total RNA was extracted from patients’ samples and from a pool of healthy subjects by using the mirVana™ miRNA Isolation Kit (Thermo Fisher Scientific, Waltham, MA, USA) and the RNeasy MinElute Cleanup Kit (Qiagen, Venlo, The Netherlands) according to the manufacturer’s instructions. RNA was retro-transcribed and the expression of BCL2, PLCB1 and PLCG1 genes was assessed using a standard TaqMan Real-Time PCR method (Applied Biosystems, Foster City, CA, USA), with the Glyceraldehyde 3-phosphate dehydrogenase TaqMan probe as the endogenous control [54] and a pool of healthy subjects as an internal control, as previously reported [55].

### GEO dataset analysis

For further validation, the Gene Expression Omnibus (GEO) database was analyzed. The publicly available GSE107400 dataset was used to validate miR-192 gene expression (probe PSR11022915.hg.1) in a larger MDS cohort, as it is generated by gene expression data from 176 MDS patients and 20 healthy subjects using the Affymetrix Human Transcriptome Array 2.0 platform [40]. The publicly available GSE4619 and GSE19429 datasets [41, 42], using the Affymetrix GeneChip Human Genome U133 Plus 2.0 arrays, were used to validate BCL2 gene expression (probe 207005_s_at). The GSE4619 dataset contains data obtained from 55 patients with MDS and 11 healthy subjects, while the GSE19429 dataset includes data from 183 patients with MDS and 17 healthy subjects.

### miRNA extraction, microarray and expression

RNA was isolated from MNCs of MDS patients treated with AZA + LEN and concentrated by using the mirVana™ miRNA Isolation Kit (Thermo Fisher Scientific) and the RNeasy MinElute Cleanup Kit (Qiagen) according to the manufacturer’s instructions. The RNA samples were biotin-labeled using the FlashTag™ Biotin HSR RNA Labeling Kit (Thermo Fisher Scientific), hybridized on the Affymetrix GeneChip miRNA 4.0 array, and then scanned with a Scanner 3000 according to the manufacturer’s protocols (Affymetrix, Inc., Santa Clara, CA, USA). Data analysis was performed by adopting an open-source R-bioconductor pipeline (https://bioconductor.org/). Briefly, the raw data (stored on CEL files) were processed by Robust Multichip Average method (package oligo, function rma) to perform background correction, quantile normalization across array and the calculation of probe-level intensities. Each probe was annotated by the package pd.mirna.4.0. Then, lmFit and eBayes functions (from limma package) were applied, respectively, to fit the linear model and to compute the moderate t-statistics in order to compare the miRNA expression profile of samples at baseline (T0) versus the 4th cycle (T4), T4 versus the 8th cycle (T8) and T0 versus T8.

For each differentially expressed miRNA, experimentally validated miRNA-target interactions were sought using miRPathDB 2.0 and KEGG (Release 94.0, April 1, 2020).

To examine and validate the expression of miR-192-5p, miR-21-5p and miR-224-5p on MDS patients, reverse transcription was performed using the Advanced miRNA cDNA Synthesis Kit (Applied Biosystem) and the ABI PRISM 7500 (Applied Biosystems) instrument, using a pool of healthy subjects as internal reference and miR-16-5p probe as endogenous control.

#### Tissue cell cultures

Monocytic THP-1 cell line (ATCC, Manassas, VA, USA) was grown at 37 °C with 5% CO2 in RPMI 1640 Medium (Corning, Manassas, VA, USA) supplemented with 10% heat-inactivated fetal bovine serum and 1% L-glutamine (Corning) at an optimal cell density of 0.3 × 10^6^–0.8 × 10^6^ cells/mL.

#### Cell transfection and luciferase assay

THP-1 cells were seeded at a density of 150 × 10^4^ cells/mL in 24-well plates. Transient transfection was carried out by using Lipofectamine RNAiMAX (Thermo Fisher Scientific) combined with 200 ng of 3′-UTR BCL2 plasmid (Genecopoeia, Rockville, MD, USA) and 30 nM mirVana™ miRNA Mimic (Thermo Fisher Scientific) in serum-free medium, according to the manufacturer’s protocols.

Luciferase assay was evaluated on THP-1 co-transfected cells. After 24 h of incubation, the activities of Firefly and Renilla luciferases were measured through the Luc-Pair™ Duo-Luciferase Assay Kit (Genecopoeia) following the manufacturer’s instructions. All experiments were carried out at least in duplicate and repeated at least four times.

### Statistical analyses

All statistical analyses were performed by using the GraphPad Prism Software. (v.5.0, La Jolla, CA, USA), and categorical data were expressed as percentages. Student’s *t* test with Dunnett’s and Bonferroni post-analysis tests of variance were used to compare continuous values. Tests were considered statistically significant when the *p* value was < 0.05. Survival analyses were done according to the Kaplan–Meier method and compared using the Log-rank test, while the Cox proportional hazard model was used to estimate hazard ratios (HR) and 95% confidence intervals (95% CI). ROC curves were used to evaluate the accuracy of miR-192-5p quantification to correlate with the response to AZA + LEN therapy in our MDS patients. Sensitivity, specificity, and the area under the curve (AUC) were calculated; *p* values < 0.05 were statistically significant.

## Supplementary Information

**Additional file 1**. Tables S1–S6.

**Additional file 2**. Figure S1.

**Additional file 3**. Supplementary materials and methods.

## Data Availability

Publicly available datasets from NCBI Gene Expression Omnibus (GEO) profiles were used: GSE114922, GSE4619, GSE114869. All other data generated or analysed during this study are included in this article (and its supplementary information files).
