# Supplementary material for: A miRNA screening identifies miR-192-5p as associated with response to azacitidine and lenalidomide therapy in myelodysplastic syndromes
Source: Clin Epigenetics. 2023 Feb 20;15:27. doi: 10.1186/s13148-023-01441-9 (PMC9940408; doi:10.1186/s13148-023-01441-9)
Supplement: Supplementary file 2 — Additional file 2. Figure S1. [file 13148_2023_1441_MOESM2_ESM.pdf]

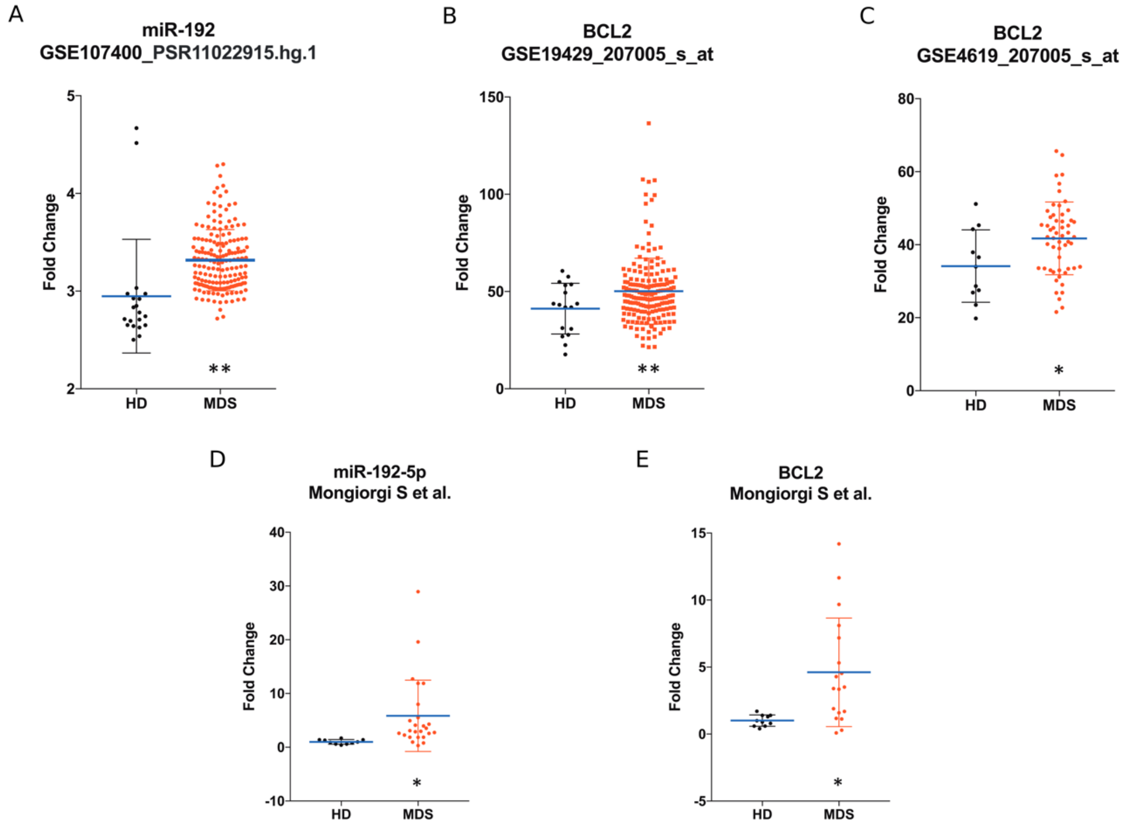

Supplementary Figure 1. Gene Expression Analysis of miR-192 and BCL2 in gene expression datasets. **(A)** Gene expression of miR-192 (probe PSR11022915.hg.1), in MDS patients (MDS) and healthy subjects (HD). Data were obtained from the GSE107400 dataset. The blue line indicates the mean amount of miR-192 gene expression. The gene up-regulation in MDS is statistically significant. **p<0.01 vs HD. **(B)** Gene expression of BCL2 (probe 207005_s_at), in MDS patients (MDS) and healthy subjects (HD). Data were obtained from the GSE19429 dataset. The blue line indicates the mean amount of BCL2 gene expression. The gene up-regulation in MDS is statistically significant. **p<0.01 vs HD. **(C)** Gene expression of BCL2 (probe 207005_s_at), in MDS patients (MDS) and healthy subjects (HD). Data were obtained from the GSE4619 dataset. The blue line indicates the mean amount of BCL2 gene expression. The gene up-regulation in MDS is statistically significant. *p<0.05 vs HD. **(D)** Gene expression of miR-192-5p in our patients' cohort (MDS) as compared with our group of healthy subjects (HD). The blue line indicates the mean amount of miR-192-5p gene expression. The gene up-regulation in MDS is statistically significant. *p<0.05 vs HD. **(E)** Gene expression of BCL2 in our patients' cohort (MDS) as compared with our group of healthy subjects (HD). The blue line indicates the mean amount of BCL2 gene expression. The gene up-regulation in MDS is statistically significant. *p<0.05 vs HD.
