# Supplementary material for: A miRNA screening identifies miR-192-5p as associated with response to azacitidine and lenalidomide therapy in myelodysplastic syndromes
Source: Clin Epigenetics. 2023 Feb 20;15:27. doi: 10.1186/s13148-023-01441-9 (PMC9940408; doi:10.1186/s13148-023-01441-9)
Supplement: Supplementary file 3 — Additional file 3. Supplementary materials and methods. [file 13148_2023_1441_MOESM3_ESM.pdf]

SUPPLEMENTARY INFORMATION: MATERIALS AND METHODS

Patient Characteristics. All samples came from several Italian hematological centers and were centralized at the Institute of Hematology "L. and A. Seràgnoli", Policlinico Sant'Orsola–Malpighi Hospital, Bologna, Italy. The hematological centres involved were: the Haematology and Haematopoietic Stem Cell Transplant Center, S.Salvatore Hospital, Pesaro, Italy; the Hematology Unit, Arcispedale Santa Maria Nuova IRCCS, Reggio Emilia, Italy; the Division of Hematology, Guglielmo da Saliceto Hospital, Piacenza, Italy; the Hematology Unit, Hospital Santa Maria delle Croci, Ravenna, Italy; the Division of Hematology and Bone Marrow Transplantation, Udine, Italy; the Hematology and BMT Center, Department Medicine and Surgery, University of Parma, Parma, Italy; the Department of Medical and Surgical Sciences, University of Modena and Reggio Emilia, Modena, Italy; the Hematology Section, Department of Medical Sciences, University of Ferrara, Ferrara, Italy; and the Hematology Unit, Infermi Hospital, Rimini, Italy. The MDS diagnosis was defined according to the World Health Organization (WHO) classification (1) and, according to the Revised International prognostic scoring system (R-IPSS) (2), patients were divided into subgroups by risk: intermediate risk (n=4), high-risk (n=12), very high risk (n=10). However, throughout the text, all patients with MDS are defined as high-risk MDS.

Patient treatment and evaluation of response. Patients were treated with Azacitidine (75 mg/m2/die for 7 days every 28 days) and Lenalidomide (10 mg/day, arm 1: days 1-21 or arm 2: 6-21 and randomly assigned to arm 1 or arm 2, orally) every 4 weeks. The induction treatment was planned for 8 cycles. For responder patients this schedule was continued until disease progression or unacceptable toxicity. The response to treatment and the clinical outcome were evaluated according to the revised International Working Group (IWG) response criteria (3). Patients were considered evaluable if they completed at least 6 cycles of therapy or showed either a positive response or disease progression before the 6th cycle (T6). We also recorded the time to AML evolution (calculated from the date of diagnosis according to the WHO classification (1) , i.e. >20% marrow blasts), survival and causes of

death. Data were censored when patients died or were lost during follow-up. Patients who achieved a complete remission (CR), partial remission (PR), or any hematologic improvement (HI), according to the revised IWG criteria (3), were considered responders, whereas all the other outcomes were defined as non responders. The duration of response was assessed in patients who showed a clinical response to treatment.

REFERENCES

1. Vardiman JW, Harris NL, Brunning RD. The World Health Organization (WHO) classification of the myeloid neoplasms. Blood. 2002;100(7):2292-302.

2. Greenberg PL, Tuechler H, Schanz J, Sanz G, Garcia-Manero G, Sole F, et al. Revised international prognostic scoring system for myelodysplastic syndromes. Blood. 2012;120(12):2454-65.

3. Cheson BD, Greenberg PL, Bennett JM, Lowenberg B, Wijermans PW, Nimer SD, et al. Clinical application and proposal for modification of the International Working Group (IWG) response criteria in myelodysplasia. Blood. 2006;108(2):419-25.
